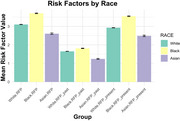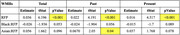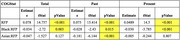# Exploring Racial Disparities in Cognitive Decline and Dementia: Analyzing Total, Past, and Present Risk Factors Profiles, Cognition, and Vascular Brain Health Outcomes

**DOI:** 10.1002/alz70860_105132

**Published:** 2025-12-23

**Authors:** Tamara Khudair, Mahsa Dadar, Cassandra Morrison

**Affiliations:** ^1^ Carleton University, Ottawa, ON, Canada; ^2^ Douglas Mental Health University Institute, Montréal, QC, Canada; ^3^ Department of Psychiatry, McGill University, Montréal, QC, Canada

## Abstract

**Background:**

Dementia affects millions worldwide, with modifiable risk factors contributing to cognitive decline and poor brain health. However, there is limited research exploring how these factors influence cognition and brain structure if they occur during midlife or late life. Furthermore, given the racial disparities in risk factors, more research is needed to investigate how race interacts with risk factors in midlife and late life to impact cognition and the brain.

**Method:**

This study included data from 35,513 participants, including 28,944 White, 5,578 Black, and 991 Asian from the National Alzheimer's Coordinating Center (NACC) large open‐access dataset. Linear regression models were conducted to assess the associations between total, past, present risk factor profiles (RFPs), race (White, Black, Asian), cognition, and vascular brain health outcomes (white matter hyperintensities [WMH], infarcts). Models included age, sex, education, and clinical diagnosis as covariates.

**Result:**

WMHs were significantly associated with total, past, present RFPs in both White and Black participants (*p* < 0.001). However, Asian participants exhibited less change in WMHs compared to Whites, particularly in response to past RFPs (*p* < 0.05). Similarly, infarcts were significantly associated with total, past, present RFPs in White and Asian participants (*p* < 0.001), while Black participants showed less change in infarcts compared to Whites across both RFP time points (*p* < 0.05). Clinical judgement of cognitive functioning (COGSTAT) scores were inversely correlated with total, past, present RFPs (*p* < 0.001), with Asian participants showing less change in COGSTAT scores compared to White participants in response to past RFPs (*p* < 0.001).

**Conclusion:**

These findings highlight the impact that both midlife and late life risk factor exposures on late‐life brain health and underscore racial disparities. They emphasize the need for targeted interventions to mitigate cognitive decline and reduce racial disparities in dementia prevalence.